# Chronic Stress Facilitates the Development of Deep Venous Thrombosis

**DOI:** 10.1155/2015/384535

**Published:** 2015-10-20

**Authors:** Tao Dong, Yu-Wen Cheng, Fei Yang, Pei-Wen Sun, Chen-Jie Zhu, Li Zhu, Guo-Xing Zhang

**Affiliations:** ^1^Department of Physiology, Medical College of Soochow University, Suzhou 215003, China; ^2^Cyrus Tang Hematology Center, Collaborative Innovation Center of Hematology, MOH Key Lab of Thrombosis and Hemostasis, Jiangsu Institute of Hematology, The First Affiliated Hospital, Soochow University, Suzhou 215003, China

## Abstract

The increasing pressure of modern social life intensifies the impact of stress on the development of cardiovascular diseases, which include deep venous thrombosis (DVT). Renal sympathetic denervation has been applied as one of the clinical approaches for the treatment of drug-resistant hypertension. In addition, the close relationship between oxidative stress and cardiovascular diseases has been well documented. The present study is designed to explore the mechanism by which the renal sympathetic nerve system and the oxidative stress affect the blood coagulation system in the development of DVT. Chronic foot shock model in rats was applied to mimic a state of physiological stress similar to humans. Our results showed that chronic foot shock procedure could promote DVT which may be through the activation of platelets aggregation. The aggravation of DVT and activation of platelets were alleviated by renal sympathetic denervation or antioxidant (Tempol) treatment. Concurrently, the denervation treatment could also reduce the levels of circulating oxidation factors in rats. These results demonstrate that both the renal sympathetic nerve system and the oxidative stress contribute to the development of DVT in response to chronic stress, which may provide novel strategy for treatment of clinic DVT patients.

## 1. Introduction

Deep venous thrombosis (DVT) is a common cardiovascular disease associated sequelae, pulmonary embolism, which is the third most common cause of death from cardiovascular diseases after heart attack and stroke. A slow blood flow, vein wall damage, and a hypercoagulable state are the three principal risk factors for DVT development. Consequently, anticoagulants and thrombolytics are the two main treatments for clinical DVT patients. The triad of risk factors predisposing to thrombus formation, postulated by Virchow, includes changes in the ratio between blood components, the integrity of the vessel wall, and the blood flow rate. The blood coagulation system plays a key role in protecting mammals against lethal bleeding. In all forms of thrombosis, coagulation and inflammation are the two principal pathways acting together to coordinate the body's responses to injury [1, 2].

Over the past few decades, psychological factors, such as stress and depression, have been recognized as important factors affecting human health [3]. Long periods of anxiety will induce the development of cardiovascular diseases. Moreover, by occurring simultaneously, depression and anxiety will aggravate the development of cardiovascular disease even further. In addition, stress and other psychological factors have been demonstrated to be closely related to the occurrence of stroke and myocardial infarction [4]. Numerous studies also have shown that stress can cause long-lasting structural damage to tissues and organs [5]. The chronic electric foot shock procedure has been characterized as a model of uncontrollable and unpredictable psychological stress [6], which has been demonstrated to be able to induce increase in systolic blood pressure [7–9]. However, there is still no report related to the effects of chronic shock on the development of DVT.

The role of sympathetic renal nerve in the development of hypertension has been demonstrated in both experimental and clinical observations [10, 11]. There are two types of sympathetic renal nerve: renal afferent nerves and renal efferent nerve. The afferent sympathetic fibers originate from the kidneys, and by modulating central sympathetic outflow they directly modify neurogenic hypertension. At the same time, the efferent nerve enhances sodium and water retention, stimulates renin release, and alters renal blood flow [10–13]. In this way, both short-term and long-term blood pressure could be influenced by renal sympathetic nerve [13]. Clinical studies have reported the beneficial effects of renal sympathetic denervation in patients with refractory hypertension [9]. In addition to the lowering effect of denervation on blood pressure, additional benefits have also been reported in cardiovascular diseases, diabetes [9], renal dysfunction, cardiac hypertrophy [1], heart failure [9], and arrhythmias [14]. Hypertension, a major risk factor for many diseases, can increase endothelial dysfunction and promote thrombosis and is also closely related to the incidence of cardiocerebral vascular diseases [9]. Therefore, renal sympathetic denervation may provide new strategy in prevention and treatment of cardiovascular diseases under high stress condition.

Recently, experiments show that oxidative stress might be responsible for the change in endothelial function [15]. The increased reactive oxygen species produced by vascular endothelium and circulating blood cells will impair vasomotor and endothelial barrier functions and enhance thrombus formation [16]. Oxidative stress was also found to be a determinant of platelet activation [17], which was the risk factor for atherothrombosis. However, whether chronic stress could affect coagulation system by increasing of oxidative stress is still unknown.

Currently, no reports are directly linking psychological stress with the coagulation system and cardiocerebral vascular diseases. Considering that the activation of the coagulation system has an important influence on both physiological hemostasis and pathological thrombosis [9], we applied foot shock stress model in rats to explore whether chronic could affect the development of DVT and the possible mechanisms involved.

## 2. Materials and Methods

### 2.1. Animal Preparation

Ten-week-old male Sprague-Dawley rats, obtained from Shanghai Laboratory Animal Center, were used in this study. Animals were maintained in a 25°C temperature-controlled environment with a 12 : 12-hour light : dark cycle. Rats exposed to the stress protocol were individually placed into a foot shock stress box, where they received a 4-hour session of electrical foot shock through an electrified grid floor delivering a 5-second long 0.15 mA shock every 30 seconds. The rat renal sympathetic nerve was surgically severed while animals were under a 10% chloral hydrate-induced anesthesia. After a one-week recovery period, the foot shock protocol was started. Tempol (10 mg/kg/day) was administered by intraperitoneal injection after the start of the stress protocol [18–20]. Venous thrombosis was induced under anaesthetized conditions with 10% chloral hydrate as previously described by Leung [21]. Briefly, the abdomen was opened, and the inferior vena cava (IVC), after being carefully separated from the surrounding tissues, was ligated tightly just below the left renal vein using a cotton thread. Then, the abdomen was closed with a double layer of sutures, closing the peritoneum with muscles first and then the skin separately. After twelve hours, the animals were anaesthetized again, the abdomen was reopened, and the plasma and thrombus were collected for further analysis [2]. The present study was performed in conformity with the European Convention for the Protection of Vertebrate Animals used for Experimental and Other Scientific Purposes (Council of Europe number 123, Strasbourg, 1985). All surgical procedures were approved by the Soochow University and performed in accordance with the guidelines for the care and use of animals established by the Soochow University.

### 2.2. Plasma Corticosterone Levels Measurements

Plasma corticosterone levels were measured using a commercially available enzyme-linked immunosorbent assay (ELISA) kit (TSZ Elisa, USA).

### 2.3. Thrombus Weight Measurements

From the reopened abdominal cavity, the ligated segment of the vena cava was removed and opened longitudinally to remove the formed thrombus, which was rinsed and weighed on filter paper.

### 2.4. Analysis of the Blood Coagulation Parameters

Prothrombin time (PT), activated partial thromboplastin time (APPT), and thrombin time (TT) were measured using an automated blood coagulation analyzer (Sysmex Corporation CA-50, Japan). From the reopened abdominal cavity, blood (4.5 mL) was collected from the inferior vena cava using a disposable syringe, containing 0.5 mL of a 3.8% sodium citrate solution, and transferred into autoclaved centrifuge tubes. Half of the blood was centrifuged at 3000 rpm for 10 min and the serum was collected. A 0.1 mL serum aliquot was combined to 0.1 mL of PT reagent. After preheating for 20 min, the PT was measured using an automated blood coagulation analyzer, as mentioned above. The APPT and the TT were measured using the same method as the PT.

Platelet aggregation was measured using a platelet aggregation analyzer (Chrono-Log 560 Ca, Germany). After the cavity was reopened, 4.5 mL blood was collected from the inferior vena cava by using a disposable syringe contained with 0.5 mL sodium citrate (3.8%) and then transferred to centrifuge tube. The second half of the blood was centrifuged at 1000 rpm for 10 min to obtain the platelet-rich plasma. The blood remaining in the tube was centrifuged at 3000 rpm for 10 min to prepare the platelet-poor plasma. Then, coagulation of the plasma samples was stimulated using collagen protein and adenosine diphosphate disodium (ADP) (1 mM, 10 mL) as platelet agonists.

### 2.5. Determination of Plasma Noradrenaline (NA) Concentration

Plasma noradrenaline (NA) levels were measured using a commercially available enzyme-linked immunosorbent assay (ELISA) kit (TSZ Elisa, USA).

### 2.6. Measurement of Lipid Peroxidation Levels and Plasma Superoxide Dismutase (SOD) and Glutathione Peroxidase (GSH-Px) Activity

Plasma SOD and GSH-Px activity and lipid peroxidation levels (thiobarbituric acid reactive substances, TBARS) were measured using commercially available enzyme-linked immunosorbent assay (ELISA) kits (TSZ Elisa, USA).

### 2.7. Statistical Analyses

All of the data were presented as the mean ± SEM. The statistical significance of the comparisons between more than two groups was tested using a two-way ANOVA followed by the Newman-Keuls test or using an unpaired two-tailed Student's *t*-test. *P* values < 0.05 were considered statistically significant.

## 3. Results

### 3.1. Effect of Shock, Denervation, and Tempol Treatment on the Plasma Corticosterone Concentrations

**Figure 1:**
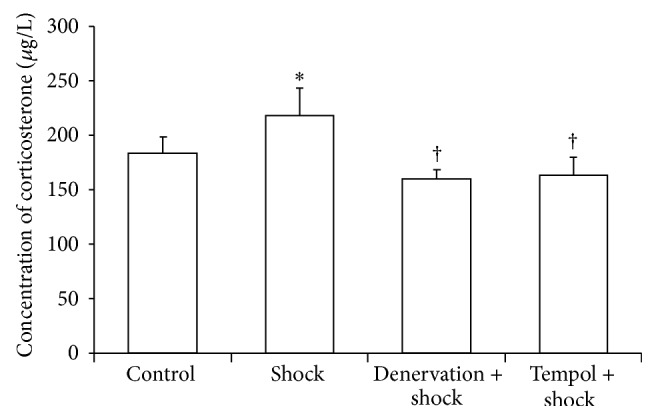
Plasma concentrations of corticosterone. Concentrations of corticosterone in plasma in each group after two-week stress were measured as described in Materials and Methods section. Data of each group (*n* = 15) were presented as mean ± SEM. ^*^ *P* < 0.05 compared with control group. ^†^ *P* < 0.05 compared with stress group.

Plasma corticosterone levels (the marker of stress) were markedly increased in the foot shock group when compared with the control group (*P* < 0.05, Figure 1) and were markedly suppressed in both the denervation plus shock and the Tempol plus shock groups compared with the foot shock alone group (*P* < 0.05, Figure 1). This result indicated that chronic foot shock significantly increased plasma corticosterone when the body was under stress, and both of denervation and Tempol treatment could alleviate the stress state.

### 3.2. Effect of Shock, Denervation, and Tempol Treatment on the Weight of IVC Ligation-Induced Thrombi

**Figure 2:**
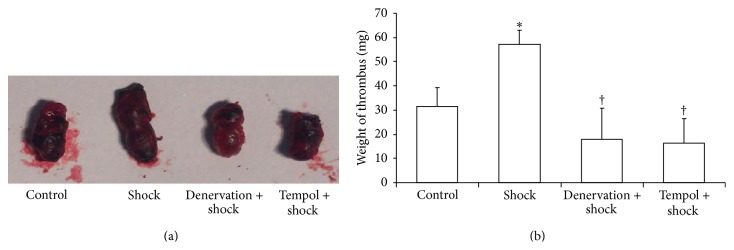
Weight of thrombus. (a) Representative image of thrombus in each group. (b) Weight of thrombus was measured in each group after two-week stress as described in Materials and Methods section. Data of each group (*n* = 15) were presented as mean ± SEM. ^*^ *P* < 0.05 compared with control group. ^†^ *P* < 0.05 compared with stress group.

Thrombi were collected 12 hours after IVC ligation and weighted. In the foot shock group, the thrombus weight was significantly increased compared with that of the control group (*P* < 0.05, Figure 2). However, the thrombus weight of the denervation plus shock and Tempol plus shock groups remained unchanged compared with the control group thrombi but was significantly decreased compared with the foot shock group thrombi (*P* < 0.05, Figure 2). These results suggest that chronic shock could facilitate the formation of DVT, while both denervation and Tempol treatment could inhibit stress-induced increase of DVT formation.

### 3.3. Effect of Shock, Denervation, and Tempol Treatment on Blood Coagulation Parameters

**Figure 3:**
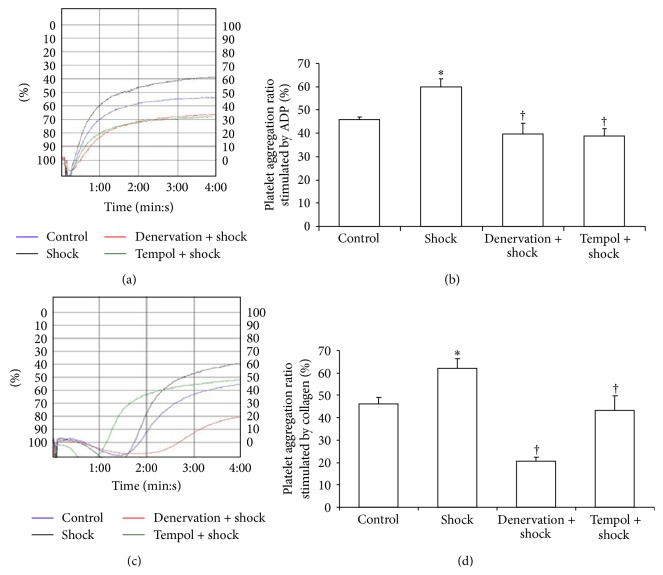
Platelet aggregation rate. Platelet aggregation rate stimulated by ADP (a, b) and platelet aggregation rate stimulated by collagen (c, d) were measured in each group after two-week stress as described in Materials and Methods section through platelet aggregation analyzer. (a) and (c) represent platelet aggregation trace provided by platelet aggregation analyzer. Data of each group (*n* = 15) were presented as mean ± SEM. ^*^ *P* < 0.05 compared with control group. ^†^ *P* < 0.05 compared with stress group.

After blood collection from the IVC, blood parameters (PT, APPT, TT, and platelet aggregation) were measured. There was a significant difference in the PT, TT, and platelet aggregation parameters between the control and the foot shock groups. The PT of the foot shock group was lower than that of the control group; however, the TT and platelet aggregation parameters were higher in the foot shock group compared with the control group. In parallel, a significant decrease was observed in the TT and platelet aggregation parameters of the denervation plus shock and Tempol plus shock groups compared with the foot shock group (*P* < 0.05, Table 1 and Figure 3). These results reveal that chronic shock could enhance coagulation system by activation of platelet aggregation.

### 3.4. Effect of Shock, Denervation, and Tempol Treatment on the Plasma Noradrenaline (NA) Concentrations

**Figure 4:**
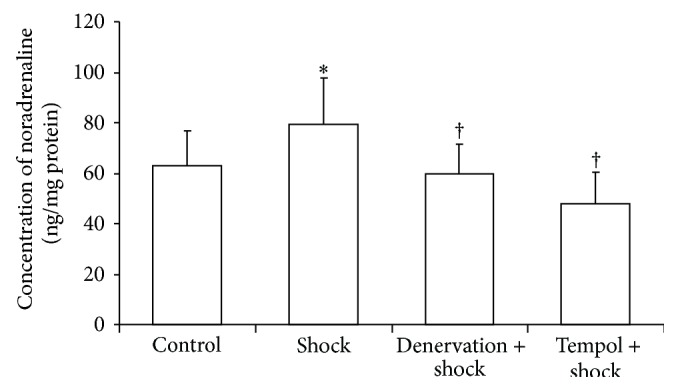
Plasma concentrations of noradrenaline. Concentrations of noradrenaline in plasma in each group after two-week stress were measured as described in Materials and Methods section. Data of each group (*n* = 15) were presented as mean ± SEM. ^*^ *P* < 0.05 compared with control group. ^†^ *P* < 0.05 compared with stress group.

Foot shock significantly increased the plasma noradrenaline (NA) levels compared with control group (*P* < 0.05, Figure 4). The plasma NA levels in the denervation plus shock and Tempol plus shock group were significantly suppressed compared with the foot shock group (*P* < 0.05, Figure 4). These results confirm the success of the renal denervation surgical procedure.

### 3.5. Effect of Shock, Denervation, and Tempol Treatment on Plasma SOD and GSH-Px Activity and on TBARS Levels

**Figure 5:**
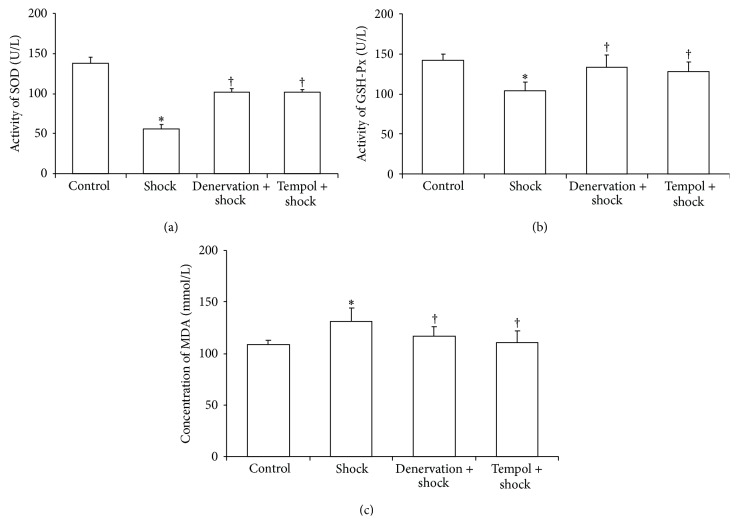
Plasma SOD, GSH-Px activities, and TBARS levels. Plasma SOD activity (a), GSH-Px activity (b), and TBARS levels (c) in each group after two-week stress were measured as described in Materials and Methods section. Data of each group (*n* = 15) were presented as mean ± SEM. ^*^ *P* < 0.05 compared with control group. ^†^ *P* < 0.05 compared with stress group.

The plasma SOD activity in the stress group was markedly reduced compared with the control group (*P* < 0.05, Figure 5(a)). The plasma SOD activity in the denervation plus shock and Tempol plus shock groups was markedly elevated compared with the foot shock group (*P* < 0.05, Figure 5(a)).

The plasma GSH-Px activity in the foot shock group was also markedly higher than that of the control group (*P* < 0.05, Figure 5(b)). The plasma GSH-Px activity in the denervation plus shock and Tempol plus shock groups was markedly elevated compared with the foot shock group (*P* < 0.05, Figure 5(b)).

Foot shock led to a marked increase in the levels of plasma thiobarbituric acid reactive substances (TBARS) compared with control group (*P* < 0.05, Figure 5(c)). The plasma TBARS levels in the denervation plus shock and Tempol plus shock groups were markedly suppressed compared with the foot shock group (*P* < 0.05, Figure 5(c)).

## 4. Discussion

In this study, we identified that DVT formation is facilitated under stress conditions and that changes in the blood coagulation system are induced by stress. Accumulating data have shown that chronic psychological stress activated two systems: one is hypothalamus-pituitary-adrenal cortex (HPA) system which was mainly mediated by release of catecholamine, cortisol, vasopressin, endorphins, and aldosterone [7, 22]; the other is through activation of sympathetic-adrenal medullar system [22–25]. As shown in the data, the corticosterone levels of chronic shock group were significantly increased, indicating that HPA was activated. What is the role of sympathetic nerve system in response to stress? Since it has been demonstrated that renal sympathetic system activation is highly related to the development of hypertension, and renal denervation is a new treatment in clinical for refractory hypertension patients [26], renal sympathetic system must play an important role in cardiovascular diseases. In addition, recent observations show that high stress condition is highly related to cardiovascular diseases [4] especially cardiac and brain infarction; therefore, we speculate whether chronic stress will activate coagulation system through activation of renal sympathetic nerve system. In the present study, we firstly found that chronic stress could aggregate the DVT formation, suggesting that stress will increase the risk of cardiovascular diseases. In addition, we measured parameters defining the activity of the blood coagulation system, including PT, APPT, TT, and platelet aggregation. Our data showed that there was a significant change in platelet aggregation induced by both ADP and collagen suggesting that the aggravating effect of stress on DVT formation was mediated by activation of platelet. Our results firstly reveal the mechanism which links the high stress condition and cardiovascular diseases. Although our present data clearly demonstrated the role of renal sympathetic nerve system in the chronic shock induced development of DVT, it is hard to identify whether afferent or efferent nerve plays a major role due to the limitation of surgical procedure. We speculate that both are involved in; the reason is that afferent nerve could affect neurogenic control of blood pressure which may contribute to the development of DVT; and efferent nerve could regulate renal secretion of noradrenaline which may also contribute to the development of DVT.

The presence of damaged endothelium and activated clotting factors or platelets facilitates the development and progression of DVT [27]. Platelet aggregation (i.e., when platelets adhere to each other) occurring at sites of vascular injury has long been recognized as critical for thrombosis development [28]. In the present study, we focused mainly on platelet function in DVT. The phenomena of platelet adhesion, release, or aggregation are also known as platelet activation [29]. Activated platelets play an important role in the thrombosis process. Our data show that platelet aggregation increased after stress treatment, along with the enhancement in DVT formation. The platelet count was used to normalize the measure of platelet activity; however, no statistics on the number of platelets in each group were performed.

Our analyses of the plasma GSH-Px and SOD activity, as well as the plasma TBARS level, showed that the body is in a state of oxidative stress induced by chronic foot shock treatment, which was inhibited by renal denervation. Several reports have shown that NAD(P)H oxidase activity could be directly increased by the *α*1- and *β*2-receptors [30] through the catecholamines (CA) released by the renal sympathetic nerve. Moreover, *β*1-receptor antagonists were shown to reduce the vascular oxidative stress caused by NAD(P)H oxidase activation [31]. Therefore, we can say that the renal sympathetic innervation directly increases oxidative stress levels. It has been reported that platelet aggregation, an additional risk factor for thrombus, is associated with oxidative stress [32]. Oxidative stress could directly increase platelet aggregation through oxygen-free radicals located on the platelet surface [32]. Solid evidence has demonstrated that oxidative stress could directly activate platelets through a variety of ways. As the product of oxidative stress O^2−^ could react with platelet or endothelium, then NO derived from ONOO-, which is of particular importance for vascular thrombosis, also has such effects. Several studies have shown that O^2−^ could reduce the threshold for platelet activation to thrombin, collagen, or ADP and O^2−^ may even be able to induce spontaneous aggregation [33–35]. The activated platelet even could produce ROS; the role of this endogenous ROS is similar to exogenous ROS in platelet activation. Generally, several scenarios leading to stress-induced platelet aggregation exist. In the first one, the renal sympathetic nerve is activated by stress, which leads to an increased oxidative stress throughout the body that is then followed by an increase in platelet aggregation. In the other scenario, stress directly triggers the body oxidative stress production, which directly increases platelet aggregation. Renal sympathetic nerve denervation may affect the platelet activation directly, however, the mechanism of which remains to be explored.

Our data showed that chronic stress treatment has no significant effect on either APPT or PT, suggesting that the stress-facilitated DVT may not be associated with the extrinsic or intrinsic coagulation system. Nevertheless, chronic stress treatment could markedly increase TT, which indicates that the conversion time of fibrinogen into fibrin was prolonged because of a hyperfibrinolysis. Hence, we speculate that fibrinolytic hyperfibrinolysis was due to an enhanced blood coagulation under the condition of chronic stress. Concurrently, the renal denervation and antioxidant treatment could decrease platelet aggregation, which in turn suppressed blood coagulation. Based on our data, we could observe that the TT tended towards a normal rate in these two conditions when compared with the shock group.

It should be noted that, in our present observation, we found that Tempol treatment could reduce chronic stress-induced increase of corticosterone levels, suggesting the involvement of oxidative stress in HPA activation induced hormone release, indicating that antioxidant treatment may provide some beneficial effects in HAP activation induced organ injury. However, we did not find any difference between denervation and antioxidant treatments in any parameters, indicating that sympathetic nerve and oxidative stress may independently contribute to the development of DVT induced by chronic shock.

In conclusion, chronic stress could increase platelet aggregation directly via the activation of the renal sympathetic nerve and increase of oxidative stress. Then, DVT comes to be facilitated by the increase in platelet aggregation. A number of studies have shown that atherosclerosis and other cardiovascular diseases are closely associated with oxidative stress [36] and that patients often present with low blood antioxidant levels [37] and enhanced levels of oxidative stress markers [38]. So, in view of the cardiocerebral vascular diseases and DVT, we can prevent and treat these diseases by targeting the therapy at the hormonal and antioxidant levels. In addition to this, it is a new way of therapy through renal sympathetic nerve.

## Figures and Tables

**Table 1:** Effect of shock, denervation, and Tempol treatment on PT, APPT, and TT.

	Control	Shock	Denervation + shock	Tempol + shock
PT (s)	11.97 ± 0.47	10.4 ± 0.15^∗^	11.03 ± 0.52	11.25 ± 0.35
APPT (s)	19.85 ± 0.14	19.05 ± 0.87	19.72 ± 1.26	18.28 ± 2.11
TT (s)	47.98 ± 0.55	64.65 ± 2.75^∗^	56.30 ± 2.41^†^	53.25 ± 3.16^†^

PT, prothrombin time; APPT, activated partial thromboplastin time; TT, thrombin time; ^∗^ *P* < 0.05 versus control group; ^†^ *P* < 0.05 versus shock group.
